# Supplementary material for: Y-Site Physical Compatibility of Numeta G13E with Drugs Frequently Used at Neonatal Intensive Care
Source: Pharmaceutics. 2020 Jul 18;12(7):677. doi: 10.3390/pharmaceutics12070677 (PMC7408039; doi:10.3390/pharmaceutics12070677)
Supplement: Supplementary file 1 [file pharmaceutics-12-00677-s001.pdf]

pharmaceutics

MDPI

Supplementary Materials: Y-site Physical Compatibility of Numeta G13E with Drugs Frequently Used at Neonatal Intensive Care

Katerina Nezvalova-Henriksen, Niklas Nilsson, Camilla Tomine Østerberg, Vigdis Staven Berge and Ingunn Tho

Table S1. Composition of Numeta G13E per activated three-in-one 300 ml bag (SmPC, Baxter).

Amino Acids	Amount (g/ 300 mL)
Alanine	0.75
Arginine	0.78
Aspartic acid	0.56
Cysteine	0.18
Glutamic acid	0.93
Glycine	0.37
Histidine	0.35
Isoleucine	0.62
Leucine	0.93
Lysine	1.03
Methionine	0.22
Ornithine	0.23
Phenylalanine	0.39
Proline	0.28
Serine	0.37
Taurine	0.06
Threonine	0.35
Tryptophan	0.19
Tyrosine	0.07
Valine	0.71
Sodium glycerol phosphate	0.98
Potassium acetate	0.61
Magnesium actetate tetrahydrate	0.10
Calcium chloride dihydrate	0.55
**Carbohydrates**	**Amount (g/ 300 mL)**
Glucose monohydrate	44.00
**Lipids**	**Amount (g/ 300 mL)**
Olive oil (80%)<br>Soy bean oil (20%)	7.50
pH (ca.)	5.5
Osmolarity ca. mOsm/L	1150

Table S2. Composition of Peditrace (SmPC, Fresenius Kabi).

**Peditrace (15 mL)**	**Amount (µg/ mL)**
Zinc	250
Copper	20
Manganese	1
Selenium	2
Fluoride	57
Iodide	1

Table S3. Composition of Soluvit (SmPC, Fresenius Kabi).

**Soluvit**	**Amount (mg/ vial)**
Thiamine mononitrate	3.1
Riboflavin sodium phosphate	4.9
Nicotinamide	40
Pyridoxine hydrochloride	4.9
Sodium pantothenate	16.5
Sodium ascorbate	113
Biotin	0.06
Folic acid	0.4
Cyanocobalamine	0.005

Table S4. Composition of Vitalipid Infant (SmPC, Fresenius Kabi).

**Vitalipid Infant (10 mL)**	**Amount (µg/ mL)**
α-Tocopherol	640
Retinol	69
Phytomenadione	20
Ergocalciferol	1
Soya bean oil	100
